# The epidemiology of septic shock in French intensive care units: the prospective multicenter cohort EPISS study

**DOI:** 10.1186/cc12598

**Published:** 2013-04-25

**Authors:** Jean-Pierre Quenot, Christine Binquet, Fady Kara, Olivier Martinet, Frederique Ganster, Jean-Christophe Navellou, Vincent Castelain, Damien Barraud, Joel Cousson, Guillaume Louis, Pierre Perez, Khaldoun Kuteifan, Alain Noirot, Julio Badie, Chaouki Mezher, Henry Lessire, Arnaud Pavon

**Affiliations:** 1 Service de réanimation médicale, Centre Hospitalier Universitaire Dijon, 14 rue Paul Gaffarel, 21970 Dijon, France; 2 Centre d'investigation clinique (INSERM CIE 1), 7 Boulevard Jeanne d'Arc, 21079 Dijon, France; 3 Service de réanimation polyvalente, Centre Hospitalier, 64 avenue du Professeur Leriche, 67504 Haguenau, France; 4 Service de réanimation Médicale, Centre Hospitalier Universitaire-Nouvel Hôpital Civil, 1 Place de l'Hopital, 67000 Strasbourg, France; 5 Service de réanimation médicale, Centre Hospitalier Universitaire Jean-Minjoz, 8 Boulevard Fleming, 25000 Besançon, France; 6 Service de réanimation médicale, Centre Hospitalier Universitaire- Hôpital Hautepierre, 1 Avenue Moliere, 67098 Strasbourg, France; 7 Service de réanimation médicale, Centre Hospitalier Universitaire- Hôpital Central, 29 Avenue du Maréchal de Lattre de Tassigny, 54000 Nancy, France; 8 Service de réanimation médicale, Centre Hospitalier Universitaire, 45 Rue Cognacq Jay, 51092 Reims, France; 9 Service de réanimation polyvalente, Centre Hospitalier, 1 Place Sainte-Croix, 57000 Metz, France; 10 Service de réanimation médicale, Centre Hospitalier Universitaire- Hôpital Brabois, 5 Rue du Morvan, 54500 Vandœuvre-lès-Nancy, France; 11 Service de réanimation médicale, Centre Hospitalier, 2A Rue Jura, 68100 Mulhouse, France; 12 Service de réanimation médicale, Centre Hospitalier, 2 Rue René Heymes, 70000 Vesoul, France; 13 Service de réanimation polyvalente, Centre Hospitalier, 14, rue de Mulhouse, 90016 Belfort, France; 14 Service de réanimation polyvalente, Centre Hospitalier, 2 rue du Docteur Flamand, 25200 Montbéliard, France; 15 Service de réanimation polyvalente, Centre Hospitalier, 39 Avenue de la Liberté, 68000 Colmar, France Presented in part the at the 31st International Symposium on Intensive Care and Emergency Medicine

## Abstract

**Introduction:**

To provide up-to-date information on the prognostic factors associated with 28-day mortality in a cohort of septic shock patients in intensive care units (ICUs).

**Methods:**

Prospective, multicenter, observational cohort study in ICUs from 14 French general (non-academic) and university teaching hospitals. All consecutive patients with septic shock admitted between November 2009 and March 2011 were eligible for inclusion. We prospectively recorded data regarding patient characteristics, infection, severity of illness, life support therapy, and discharge.

**Results:**

Among 10,941 patients admitted to participating ICUs between October 2009 and September 2011, 1,495 (13.7%) patients presented inclusion criteria for septic shock and were included. Invasive mechanical ventilation was needed in 83.9% (*n *= 1248), inotropes in 27.7% (*n *= 412), continuous renal replacement therapy in 32.5% (*n *= 484), and hemodialysis in 19.6% (*n *= 291). Mortality at 28 days was 42% (*n *= 625). Variables associated with time to mortality, right-censored at day 28: age (for each additional 10 years) (hazard ratio (HR) = 1.29; 95% confidence interval (CI): 1.20-1.38), immunosuppression (HR = 1.63; 95%CI: 1.37-1.96), Knaus class C/D score *versus *class A/B score (HR = 1.36; 95%CI:1.14-1.62) and Sepsis-related Organ Failure Assessment (SOFA) score (HR = 1.24 for each additional point; 95%CI: 1.21-1.27). Patients with septic shock and renal/urinary tract infection had a significantly longer time to mortality (HR = 0.56; 95%CI: 0.42-0.75).

**Conclusion:**

Our observational data of consecutive patients from real-life practice confirm that septic shock is common and carries high mortality in general ICU populations. Our results are in contrast with the clinical trial setting, and could be useful for healthcare planning and clinical study design.

## Introduction

In recent years, our knowledge of the characteristics of patients who are admitted to critical care with sepsis, severe sepsis, or septic shock has greatly advanced thanks to the findings of numerous observational studies [1-6]. There is wide variation in the incidence of sepsis and severe sepsis in the intensive care unit (ICU) setting, with reported rates ranging from 20% to 80%, and reported mortality of 20% to 50% [1-6]. Septic shock, defined as a state of acute circulatory failure characterized by persistent hypotension unexplained by other causes, despite adequate fluid resuscitation [7], affects between 10% and 30% of patients managed in the ICU [1,3,4,8-10], and its incidence is increasing [3]. Mortality from septic shock in the ICU is estimated to range between 45% and 63% in observational studies [3], but is reportedly declining over time [3]. These differences between reports are largely related to the definitions used to define infection [4,9,11], the different phases of sepsis [7,12], and organ dysfunction [10,13,14].

In recent decades, several epidemiological studies have been published focusing on sepsis and reporting data from ICUs in France (either partially or entirely) [4,8,11,15-17]. The only French study to date to have included exclusively patients with septic shock was published by Annane et al. [3] almost 10 years ago, with data collected between 1993 and 2000. The authors of all these studies have themselves acknowledged their limitations, which include: short inclusion periods [4,8,11,15,16] that preclude any evaluation of the impact of seasons; the heterogeneity of the patients included [4,8,11,16,18]; short follow-up (for example, 2 weeks) [16]; and use of a database using ICD definitions, with the inherent risk of wrong diagnostic codes, particularly since the codes were not standardized [3]. Despite these limiting factors, the data from French ICUs is sufficiently robust to allow comparison with data from other countries. The overall frequency of septic shock was 8.2 per 100 admissions (in 2000), and crude mortality in the ICU was 60.1%, declining from 62.1% in 1993 to 55.9% in 2000 [3].

However, all these French observational studies were performed and reported before the publication of the Surviving Sepsis Campaign [7,19], and before the publication of French national guidelines for the management of sepsis published jointly by the two French scientific societies in critical care (Société de Réanimation de Langue Française (French-language society of intensive care, SRLF, and Société Française d'Anesthésie Réanimation) in 2006 [20,21]. Reports from other countries suggest that compliance with these guidelines can have a positive impact on mortality [22,23].

The objective of this study was to collect up-to-date epidemiological data from real-life practice in France on septic shock, to describe the survival probabilities at 3, 7, and 28 days after an initial episode of septic shock and to identify prognostic factors from these recent data.

## Methods

### Study population

This prospective cohort included all consecutive adult patients with a diagnosis of septic shock admitted to 14 ICUs in 10 public hospitals (5 academic teaching hospitals and 5 non-academic general hospitals) in the North-East of France, between October 2009 and September 2011. There were no specific non-inclusion criteria. Septic shock was defined based on the PROWESS-SHOCK study [24], namely documented or suspected infection requiring initiation of vasopressors despite adequate vascular filling, with at least one of the following hypoperfusion criteria: (1) metabolic acidosis (base excess ≥5 mEq/L, alkaline reserve <18 mEq/L or lactate ≥2.5 mmol/L); (2) oliguria/renal insufficiency (<0.5 mL/kg/h for 3 h or elevation >50% of baseline creatinine); or (3) hepatic dysfunction (AST or ALT >500 IU/L or bilirubin >34 μmol/L). Unlike in the PROWESS-SHOCK study, there was no minimum requirement for vascular filling in our study.

### Data collection

Data collection included: socio-demographic characteristics; chronic health status as evaluated by the Knaus score; Simplified Acute Physiological Score (SAPS) II at ICU admission [25]; SOFA score [26] over the 24 first h following vasopressor initiation; infection site and germ(s), when identified; life-support therapy in ICU and in-hospital; length of ICU and hospital stay. We also recorded immunosuppression, defined as presence of cancer (solid tumors); hematological cancer; corticoid use (>3 weeks); transplantation; acquired immune deficiency syndrome (AIDS); other (patients receiving chemotherapy; cyclophosphamides; rituximab or other anti-organ rejection agents). The Knaus Chronic Health Status score consists of: Class A: normal health status, Class B: moderate activity limitation, Class C: severe activity limitation due to chronic disease, and Class D: bedridden patient [27]. Antimicrobial therapy was classified as appropriate if the prescribed antimicrobial regimen was active against the identified pathogen. Patients were followed up until 28 days after onset of shock (or until death if death occurred first) and at hospital discharge. Patients with a second episode of shock in-hospital or who were later re-admitted for recurrent shock were not included a second time.

All data were collected using a standardized electronic case report form by dedicated clinical research assistants. Automatic checks were generated for missing or incoherent data. According to French legislation, patients (or their legal representative) were informed that their data were collected for research purposes and consent was obtained from the patient (or next of kin). Collection of nominative data was approved by the national authority for the protection of privacy and personal data, and by the ethics committee of the French Society of Intensive Care.

### Statistical analysis

Quantitative variables are reported using mean (± standard deviation (SD)) or median (Interquartile range (IQR)) according to their distribution and qualitative variables as number (percentage). The SAPS II and SOFA variables were divided into two classes according to the median, and age was divided into four categories for the estimation of survival probabilities and log-rank comparison.

Follow-up was censored at 28 days. Survival probabilities were estimated using the Kaplan-Meier product-limit method at 3, 7, and 28 days and compared using the Log rank test. At an alpha risk of 5%, a beta risk of 10%, and an expected observed mortality rate of 50%, we calculated that 1,400 patients would be necessary to ensure adequate statistical power to detect a minimal relative risk of 1.25 [28-30]. Based on conservative estimates of inclusions in participating centers, we hypothesized that a time window of 24 months would be necessary to accrue an adequate number of patients. Correlations between variables were systematically estimated using Pearson or Spearman's rank correlation, as appropriate. In case of colinearity (*P *> 0.6), the most informative variable was selected for inclusion in the model, based on clinical arguments and Akaike information criterion [31]. Multivariate analyses were performed using a Cox proportional hazards model [32] including previously selected factors associated with time to mortality, right censored at day 28 with a *P *value < 0.25 in bivariate analyses. A backward selection procedure was applied to identify factors significantly associated with time to death (*P *≤ 0.05). Proportionality was checked by testing for a non-zero slope in a generalized linear regression of the scaled Schoenfeld residuals on the natural logarithm of time [33]. The log-linearity of the relationship between continuous variables and time to death was checked using fractional polynomials [34]. Inappropriate antimicrobial therapy was considered as a time-varying covariate. All analyses were stratified by center.

Analyses were performed using SAS version 9.2 (SAS Institute, Cary, NC, USA) and Stata version 10.0.

## Results

### Study population

Patients admitted to the ICUs of participating hospitals were systematically screened between October 2009 and September 2011. A total of 10,941 patients were admitted to the participating ICUs during the study period. Among these, 1,495 (13.7%) presented a septic shock and were included in the study. Complete follow-up was obtained for 1,488 patients (99.5%); seven were lost to follow-up.

The baseline characteristics and the survival probabilities at 3, 7, and 28 days are shown in Table 1. Median age was 68 years (range, 58-78 years), almost two-thirds were men. The majority of admissions were of medical origin (84%). The most common co-morbidities were immune deficiency in 31% (*n *= 456), and 23% of patients had least two co-morbidities. The median (IQR) SAPS II and SOFA scores were 56 [45-70] and 11 [9-14], respectively. Approximately two-thirds of patients presented community-acquired infection, and more than half had respiratory tract infection (53.6%) as the primary site of infection at the origin of septic shock. The infectious organism was identified in 1,035 (69.5%) patients who presented septic shock, and an antibiogram was available in 967 of these patients (93%). Gram-negative bacilli were the most frequent pathogens in 48.7%, while Gram-positive cocci micro-organisms were identified in 35.9% (Table 2). Appropriate antimicrobial therapy, given in 898 patients; was initiated mainly before, or at the same time as septic shock (*n *= 493/860 with known time to treatment initiation), or within the 3 days following shock (*n *= 338/860). Only 69/967 (7%) patients had inappropriate antibiotic therapy.

**Table 1:** Baseline characteristics of the study population of 1,488 intensive care unit patients with septic shock, and survival probabilities at 3, 7, and 28 days (Kaplan-Meier product limit method - EPISS study - 2009 to 2011).

		Survival probabilities^a^			
			
	All (*n *= 1,488)	3 days	7 days	28 days	*P *value (log-rank test)
Age (years)					
<60	428 (28.8%)	86.0%	79.9%	67.8%	<0.0001
60 to <70	277 (25.3%)	82.2%	74.8%	57.8%	
70 to <80	437 (29.4%)	80.3%	75.0%	54.1%	
≥80	246 (16.5%)	78.0%	71.1%	45.7%	

Gender					
Female	537 (36.1%)	82.7%	76.7%	61.6%	0.04
Male	951 (63.9%)	81.7%	75.2%	55.4%	

BMI (kg/m²)					
<20	129 (8.7%)	81.4%	74.4%	55.5%	0.06
20-25	371 (24.9%)	83.0%	77.0%	56.4%	
25-30	388 (26.1%)	82.0%	77.8%	59.0%	
>30	336 (22.6%)	85.7%	79.1%	61.4%	
N/A	264 (17.7%)				

Co-morbidities^b^					
Immunosuppression^c^	459 (30.9%)	76.7%	66.6%	44.9%	<0.0001
Cancer (solid tumors)	226 (49.2%)	77.9%	68.4%	44.5%	0.97
Hematological cancer	142 (30.9%)	69.0%	57.0%	37.0%	<0.01
Corticoids	109 (23.8%)	79.8%	71.6%	52.9%	0.55
Transplantation	40 (8.7)	82.5%	77.5%	55.0%	0.15
AIDS	10 (2.2%)	80.0%	70.0%	30.0%	0.63
Other	77 (16.8%)	74.0%	64.0%	48.0%	0.90
Diabetes mellitus	387 (26.0%)	84.7%	77.7%	55.8%	0.63
Cirrhosis	133 (8.9%)	79.7%	66.8%	41.7%	<0.0001
Chronic heart failure (NYHA Class III/IV)	160 (10.8%)	78.1%	72.5%	46.1%	<0.01
Chronic respiratory failure^c^	113 (7.6%)	86.7%	81.4%	57.9%	0.80
Chronic renal failure^d^	171 (11.6%)	81.5%	75.7%	49.4%	0.04

Number of co-morbidities					
None	510 (34.3%)	85.5%	81.4%	70.0%	<0.0001
1	633 (42.5%)	80.1%	73.1%	54.7%	
2 or more	339 (23.1%)	80.6%	72.1%	44.7%	

Knaus^c^					
A/B	853 (57.4%)	85.5%	80.3%	65.0%	<0.0001
C/D	634 (42.6%)	77.4%	69.5%	47.6%	

SOFA					
≥11	823 (55.3%)	72.5%	65.6%	45.0%	<0.0001
<11	665 (44.7%)	93.8%	88.3%	73.3%	

SAPS II					
≥56	753 (50.6%)	69.3%	61.3%	40.9%	<0.0001
<56	735 (49.4%)	95.1%	90.4%	74.8%	

Site of infection^b^					
Respiratory tract	798 (53.6%)	84.1%	77.4%	56.7%	0.84
Abdominal	285 (19.2%)	83.1%	75.0%	58.2%	0.83
Renal/urinary tract	209 (14.1%)	86.6%	82.8%	69.5%	<0.001
Bloodstream	196 (13.2%)	70.4%	63.3%	49.8%	<0.001
Other	88 (5.9%)	81.8%	75.0%	57.3%	0.99

Type of infection					
Community-acquired	974 (65.5%)	82.6%	78.1%	61.9%	<0.0001
Nosocomial	514 (34.5%)	80.9%	71.2%	49.6%	

Germ identification					
Yes	1,000 (67.2%)	81.8%	75.9%	58.8%	0.21
No	488 (32.8%)	82.6%	75.4%	55.2%	

Type of admission					
Planned surgery	37 (2.5%)	89.2%	83.8%	64.2%	<0.01
Medical	1,248 (83.9%)	80.7%	74.2%	55.9%	
Emergency surgery	203 (13.6%)	89.2%	83.2%	67.3%	

Origin of patient	595 (40.0%)				
Home	23 (1.6%)	84.0%	79.1%	65.3%	<0.0001
Nursing home	870 (58.5%)	95.6%	91.3%	73.9%	
Transfer		80.3%	73.0%	51.9%	

AIDS, acquired immune deficiency syndrome; BMI, body mass index; N/A, not available; NYHA, New York Heart Association.

^a^According to the Kaplan Meier product-limit method.

^b^Patients could have more than one comorbidity and/or site of infection.

^c^One missing datum.

^‡^Two missing data.

**Table 2:** Germs responsible for infection in 1,035/1,488 septic shock patients in whom the causative microorganism was identified (EPISS study - 2009 to 2011).

Germ	All (*n *= 1,035)	Survival probabilities^a^			*P *value (log-rank test)
			
	*n *(%)	3 days	7 days	28 days	0.13
Gram-negative bacilli	505 (48.7)	81.0	74.0	59.5	
Gram-positive bacilli	4 (0.4)	0	0	66.7	
Gram-positive cocci	373 (35.9)	80.7	76.4	57.1	
Gram-negative cocci	5 (0.5)	80.0	80.0	60.0	
Fungus	33 (3.2)	90.9	81.8	47.6	
Parasite	15 (1.5)	0	93.3	73.3	
Intracellular	30 (2.9)	93.3	86.7	73.3	
Virus	22 (2.1)	90.9	86.4	63.6	
Anaerobic	33 (3.2)	75.8	72.7	54.5	
Polymicrobial	4 (0.4)	50.0	50.0	25.0	
Other	11 (1.1)	81.8	81.8	63.6	

^a^According to the Kaplan-Meier product-limit method

### Outcomes and interventions

In total, 625/1488 (42%) died within the 28 days following the septic shock. ICU and in-hospital mortality rates were 39.5% and 48.7%, respectively. Patient outcomes are described in Table 3. Life-support therapy during hospital stay is described in Table 4. Invasive mechanical ventilation was required in most patients (83.9%) at the start of septic shock. Continuous renal replacement therapy and intermittent hemodialysis were used in 32.5% and 19.6%, respectively.

**Table 3:** Outcomes at ICU discharge, at 28 days, and hospital discharge after septic shock in the study population of 1,488 patients (EPISS study - 2009 to 2011).

Outcome	All (*n *= 1,488)
ICU mortality, *n *(%)	587 (39.5%)

Median (IQR) length of ICU stay, days	9 (3-19)

28-day mortality, *n *(%)	625 (42%)

In-hospital mortality, *n *(%)	724 (48.7%)

Median (IQR) length of hospital stay, days	22 (10-43)

ICU, intensive care unit; IQR, interquartile range.

**Table 4:** Life-support therapy during hospital stay in the study population of 1,488 patients with septic shock (EPISS study - 2009 to 2011).

Treatment	*n *(%)	Median (IQR) duration (days)
Vasopressors	1,488 (100%)	4 (2-6)

Inotropes	412 (27.7%)	3 (2-6)

Invasive mechanical ventilation	1,248 (83.9%)	7 (3-14)

Non-invasive ventilation	355 (24.2%)	2 (1-4)

Continuous renal replacement therapy	484 (32.5%)	4 (2-8)

Intermittent hemodialysis	291 (19.6%)	3 (1-5)

Hydrocortisone	937 (63.0%)	N/A

Protein C	28 (1.9%)	N/A

IQR, interquartile range; N/A, not available.

### Prognostic factors

The factors found to be significantly associated with a shorter time to death, right censored at day 28, are shown in Table 5. Patients with urinary tract infection as the origin of septic shock had a significantly longer time to death (Table 5).

**Table 5:** Factors associated with time to mortality, right censored at 28 days, by the Cox model in the study population of 1,488 patients with septic shock (EPISS study - 2009 to 2011).

	Univariate Cox models		Full Cox model		Final Cox model	
	**HR (95%CI)**	***P***	**HR (95%CI)**	***P***	**HR (95%CI)**	***P***

Age (per additional 10 years)	1.19 (1.13-1.28)	<10^-4^	1.27 (1.19-1.36)	<10^-4^	1.27 (1.18-1.36)	<10^-4^
Females (*vs*. males)	0.85 (0.72-1.01)	0.062	0.99 (0.84-1.18)	0.942	--	
BMI (kg/m²)						
<20 *vs*. 20.25	1.08 (0.79-1.46)	0.641	--		--	
25-30 *vs*. 20-25	0.94 (0.76-1.18)	0.614	--		--	
>30 *vs*. 20-25	0.86 (0.68-1.09)	0.222	--		--	
Unknown *vs*. 20-25	1.34 (1.02-1.76)	0.034	--		--	
Co-morbidities						
Immunosuppression	1.71 (1.46-2.02)	<10^-4^	1.60 (1.35-1.91)	<10^-4^	1.60 (1.35-1.89)	<10^-4^
Diabetes mellitus	1.04 (0.87-1.25)	0.63	--		--	
Cirrhosis	1.60 (1.26-2.03)	<10^-4^	1.20 (0.93-1.55)	0.148	--	
Chronic heart failure (NYHA III/IV)	1.48 (1.17-1.86)	0.001	1.25 (0.96-1.63)	0.095	--	
Knaus (C/D *vs*. A/B)	1.70 (1.45-2.00)	<10^-4^	1.38 (1.15-1.65)	<10^-4^	1.49 (1.26-1.74)	<10^-4^
SOFA	1.23 (1.20-1.26)	<10^-4^	1.23 (1.20-1.27)	<10^-4^	1.24 (1.21-1.27)	<10^-4^
Site of infection						
Respiratory tract	1.00 (0.86-1.18)	0.952	--		--	
Abdominal	1.02 (0.83-1.26)	0.831	--		--	
Renal/urinary tract	0.61 (0.47-0.79)	<10^-4^	0.65 (0.49-0.85)	0.001	0.62 (0.48-0.81)	<10^-4^
Bloodstream	1.39 (1.11-1.73)	0.004	1.23 (0.97-1.56)	0.084	--	
Other	1.00 (0.72-1.41)	0.978	--		--	
Nosocomial *vs*. community infection	1.42 (1.20-1.67)	<10^-4^	1.15 (0.97-1.36)	0.102	--	
Germ identification (yes *vs*. no)	0.88 (0.75-1.04)	0.141	0.85 (0.71-1.02)	0.078	--	

Type of admission (surgery *vs*. medical)	0.68 (0.53-0.87)	0.002	--		--	
Origin (transfer *vs*. home/nursing home)	1.49 (1.25-1.77)	<10^-4^	--		--	

BMI, body mass index; CI, confidence interval; HR, hazard ratio; NYHA, New York Heart Association; SOFA, Sepsis-related Organ Failure Assessment.

To avoid colinearity between SAPS II and SOFA scores (*P *= 0.65), only SOFA score was included in the model. The origin of patients and the reason for admission were not included in the model. Factors identified by multivariate Cox analysis as significantly associated with time to death, right censored at 28 days were: age, immunosuppression, SOFA score, and Knaus C/D score (Table 5). Urinary tract infection had a significant protective effect. The hypothesis of log-linearity could not be rejected for age (*P *= 0.287) and SOFA score (*P *= 0.767). Conversely, SOFA score was shown to have a time-dependent effect (*P *< 10^-4^), with the effect decreasing over time. Inappropriate antibiotic therapy was not found to be associated with time to mortality right censored at day 28 (*P *= 0.897) (after adjusting for other covariates and for the interaction between SOFA score and the natural logarithm of time).

## Discussion

In this large-scale, multicenter study of septic shock in French ICUs, we observed an incidence of 13.5%, and death rates of 39.5%, 42%, and 48.7% at ICU discharge, 28 days, and hospital discharge, respectively. Our findings represent the most recent data on incidence and mortality of septic shock from France, using a standardized definition of septic shock [35] combined with hypoperfusion criteria, as defined in the PROWESS-SHOCK study [24]. Over the period 1993 to 2000, the overall incidence of septic shock in France was reported to be on average 8.2 per 100 admissions [3], with the authors reporting an increase over the period from 7.0 in 1993 to 9.7 per 100 admissions in 2000. Mean mortality over the same period was 60.1%, with a decreasing trend from 62.1% in 1993 to 55.9% in 2000. The increasing incidence and high mortality observed by Annane et al. was partially explained by the increasing age of patients admitted to the ICU over the period under study, with ever more co-morbidities, particularly immunosuppression.

Recent studies from various countries around the world have reported mortality rates from 35% to 59% (in-hospital or at 30 days) [5,6,8,18,36], albeit with study populations that were more heterogeneous than that included in our study. Our results are especially important in that they were prospectively collected in a broad mix of ICUs in a contemporary period over 18 months, after the publication of several major trials related to treatment of sepsis likely to have influenced management [37-39]. In these recent interventional studies, the hospital mortality rates reported in the control group ranged from 46.5% to 69% and from 30.5% to 60% in the treatment groups [37,39]. The 28-day mortality was also different in these recent interventional studies, reportedly ranging from 24% to 61% in the control group, and from 24.7% to 55% in the treatment group. The difference was explained by the inclusion and exclusion criteria, and the severity at inclusion, which may not have accurately reflected 'real life' patient populations.

In recent years, several sets of guidelines have been issued and updated on the management of sepsis in the setting of intensive care [19,23]. In addition, national guidelines have been issued in France jointly by the two French scientific societies in critical care (Société de Réanimation de Langue Française (French-language society of intensive care, SRLF, and Société Française d'Anesthésie Réanimation) in 2006 [20,21]. The implementation of these recommendations in practice has favorably influenced patient prognosis, as reported in several studies, particularly due to earlier recognition of the severity of disease, followed by consistent, multidisciplinary management [22,23,40,41]. Other authors have reported a reduction in mortality in-hospital or at 28 days, after the rigorous implementation of such guidelines [41,42]. Our data show that mortality in the ICU decreased by approximately 17% between 2000 [3] and the period 2009 to 2011 (inclusion period of our study), for patients with septic shock and comparable severity at admission (mean SAPS II score of 56 in the study by Annane et al. *vs*. 58 in our study). These data suggest that management has improved over the last decade, and undoubtedly, the publication of international clinical practice guidelines for management contributed to this trend, although a recent study by Leone et al. showed that there is still room for considerable improvement before guidelines are fully implemented [43].

Conversely, overall hospital mortality only decreased by around 10% over the same period, after initial ICU stays of 15.2 days on average in the report by Annane et al. [3]*versus *9 days in our study. This suggests that despite earlier recognition and management, with likely more appropriate therapy, the effectiveness of post-ICU care of septic shock patients remains suboptimal [44]. It is possible that certain patients expressed their desire not to be resuscitated or re-admitted to ICU, or that a decision to limit or withdraw therapy may have been made by physicians. Such factors could also explain the reduced mortality benefit that we observed during the ICU stay. Padkin et al. reported post-ICU mortality of 12.3%, corresponding to 18% of patients discharged alive from the ICU but who subsequently died before being discharged from the hospital [36]. Inappropriately early discharge [45] or discharge to an unsuitable follow-up ward because of excessive workload could also be contributing factors [45,46].

The independent prognostic factors for time to mortality right censored at day 28 identified in our study were age, immunosuppression, SOFA score, and Knaus score C/D. Conversely, we observed that urinary tract infection as the origin of sepsis had a protective effect. In a similar population, Annane et al. showed that age, severity of illness, characteristics of infection, and life-support therapy were associated with ICU mortality [3]. However, in our study, life-support therapy was not included in the multivariate analysis, as it is a time-dependent variable with no adjustment for the updated SOFA value, and this could introduce an indication bias. The prognostic factors for death in septic shock patients reported in the literature vary widely according to the type of statistical analysis (uni- or multivariate), the primary endpoint (28-day, ICU, or in-hospital mortality), and the inclusion criteria of the studies.

The rate of documented infection varies from 52% to 90% in the literature, while in our study infection with an identified microorganism was documented in nearly 60% of septic shock cases. As regards the site of infection responsible for septic shock, the most common locations were pulmonary (48.5%), abdominal (17.6%), and urinary tract (9.5%), as reported in previous studies [3,6,8,16,47]. Our results show that gram-negative organisms currently account for a majority of infections, as reported in other studies [6,8,16]. However, in our study, we did not observe the germ responsible for infection to be associated with 28-day mortality. This corroborates findings from another recent French study that included over 4,000 episodes of severe sepsis in 3,588 patients [48].

Early appropriate antibiotic therapy is of capital importance in the management of sepsis, as reported by several authors [40,49]. In our study, all patients in whom the causative microorganism was identified by antibiogram were classified according to whether they received appropriate antimicrobial therapy or not. However, it is noteworthy that in our study, antibiotic therapy (appropriate or inappropriate) was not shown to be significantly associated with time to death, right censored at 28 days, which reflects findings by other authors [50]. It is more probable that the severity, mirrored by the level of organ dysfunction at the time of the shock, and expressed by the SOFA score, is a major determinant of mortality in septic shock patients. Therefore, an organ dysfunction score should be measured at inclusion in sepsis studies, as it can be used for stratification of patients and for adjustment when assessing outcome [38]. In addition to these variables, others factors not measured in our study may also influence outcome in patients with septic shock and thus help refine prognostic prediction. For example, cytokine levels or other markers of inflammation may have a role to play, as suggested by a recent expert panel [7].

This study has several strengths. Diagnosis of septic shock was prospective and used standard criteria similar to those used in most clinical trials in this clinical setting. The sites included both university teaching hospitals and general (non-academic) hospitals of various sizes. Accrual was over a relatively long but contemporary time period, with prospective inclusion of all consecutive patients and practically no loss to follow-up. The population was homogeneous, comprising only patients with septic shock, and not a mix of sepsis, severe sepsis, and septic shock as in many published studies. The large sample size yields narrow confidence intervals around the estimates of mortality and made it possible to include a considerable number of variables in the regression analysis. Participating sites entered study data directly into a specially designed software programme, and data were of a high standard thanks to extensive data checking at the time of data entry by the clinical research assistants. Similarly, data were monitored, verified, and analyzed by a highly-qualified, central coordinating center (INSERM CIE 1).

Conversely, a few limitations of this study deserve to be underlined, and include the lack of detailed pre-ICU-admission data (for example, fluid challenge, exact time of onset of signs of sepsis). The majority of known prognostic factors were included in our analysis, but we cannot exclude that other variables not recorded in our study (for example, biomarkers) may have influenced outcome. Since selection of the investigating sites was on a voluntary basis, there is a possibility that only the most motivated centers participated, and results should not be extrapolated to other contexts. Furthermore, the fact that several participating sites were also participating in the Prowess-Shock study may have influenced prescriptions of drotrecogin alpha in our study. Lastly, the population of this study is mainly composed of medical patients (almost 84%) and therefore, results cannot be extrapolated to the entire population of ICU patients in France.

## Conclusions

In summary, our results show that a large-scale cohort of septic shock patients is feasible using simplified computer-based data collection, and shows that mortality among this patient group is still very high. This can be explained by the fact that patients with septic shock admitted to the ICU are generally older, with more co-morbidities, a worse previous state of health, and requiring more life-support therapies. These observations may be useful for quality improvement of the care provided to patients at risk of, or with confirmed septic shock, for the design of future clinical studies and for healthcare decision-makers.

## Key messages

• This is the first large-scale epidemiological study performed in France since the publication of the Surviving Sepsis Campaign recommendations and of French national guidelines for the management of septic shock.

• Mortality in the ICU among patients admitted for septic shock is declining, or rather, death occurs at a later stage. In-hospital mortality has remained constant for many years, likely due to better initial management.

• The older age, greater dependency, and more frequent co-morbidities among ICU patients admitted for septic shock probably also explain why overall mortality has remained stable over time.

• Mortality at 28 days after an initial episode of septic shock in the ICU was 42% in this prospective, multicenter, cohort study from 14 ICUs in 10 public hospitals in France. Main factors significantly associated with time to death, right censored at 28 days were age, Knaus, and SOFA scores.

## Abbreviations

ICU: Intensive Care Unit; IQR: interquartile range; SAPS II: Simplified Acute Physiological Score II; SD: standard deviation; SOFA: Sepsis-related Organ Failure Assessment.

## Competing interests

The authors declare that they have no competing interests.

## Authors' contributions

Study conception and design: JPQ, CB, AP; Data acquisition: All authors; Study coordination: VC, JC, GL, PP, AN; Statistical analysis: JPQ, CB, AP; Drafting of the manuscript: JPQ, CB, AP, FG, JCN; Critical revision of the manuscript: All; Final approval of the manuscript for submission: All.
